# MRI-Guided Regional Personalized Electrical Stimulation in Multisession and Home Treatments

**DOI:** 10.3389/fnins.2018.00284

**Published:** 2018-05-16

**Authors:** Andrea Cancelli, Carlo Cottone, Alessandro Giordani, Giampiero Asta, Domenico Lupoi, Vittorio Pizzella, Franca Tecchio

**Affiliations:** ^1^Laboratory of Electrophysiology for Translational Neuroscience, Istituto di scienze e tecnologie della cognizione (ISTC), Consiglio Nazionale Delle Ricerche (CNR), Rome, Italy; ^2^AFaR Division, Fatebenefratelli Foundation for Health Research and Education, Rome, Italy; ^3^Department of Neuroscience, Imaging and Clinical Sciences, Università degli Studi G. d'Annunzio Chieti e Pescara, Chieti, Italy; ^4^Institute for Advanced Biomedical Technologies, Università degli Studi G. d'Annunzio Chieti e Pescara, Chieti, Italy; ^5^Institute of Neurology, Catholic University of the Sacred Heart, Rome, Italy

**Keywords:** regional personalized electrode (RePE), transcranial electrical stimulation (tES), computational modeling, primary somatosensory and motor cortex (S1, M1), tDCS home treatments, non-invasive brain stimulation (NIBS)

## Abstract

The shape and position of the electrodes is a key factor for the efficacy of transcranial electrical stimulations (tES). We have recently introduced the Regional Personalized Electrode (RePE), a tES electrode fitting the personal cortical folding, that has been able to differentiate the stimulation of close by regions, in particular the primary sensory (S1) and motor (M1) cortices, and to personalize tES onto such an extended cortical district. However, neuronavigation on individual brain was compulsory for the correct montage. Here, we aimed at developing and testing a neuronavigation-free procedure for easy and quick positioning RePE, enabling multisession RePE-tES at home. We used off-line individual MRI to shape RePE via an *ad-hoc* computerized procedure, while an *ad-hoc* developed Adjustable Helmet Frame (AHF) was used to properly position it in multisession treatments, even at home. We used neuronavigation to test the RePE shape and position obtained by the new computerized procedure and the re-positioning obtained via the AHF. Using Finite Element Method (FEM) model, we also estimated the intra-cerebral current distribution induced by transcranial direct current stimulation (tDCS) comparing RePE vs. non-RePE with fixed reference. Additionally, we tested, using FEM, various shapes, and positions of the reference electrode taking into account possible small displacements of RePE, to test feasibility of RePE-tES sessions at home. The new RePE neuronavigation-free positioning relies on brain MRI space distances, and produced a mean displacement of 3.5 ± 0.8 mm, and the re-positioning of 4.8 ± 1.1 mm. Higher electric field in S1 than in M1 was best obtained with the occipital reference electrode, a montage that proved to feature low sensitivity to typical RePE millimetric displacements. Additionally, a new tES accessory was developed to enable repositioning the electrodes over the scalp also at home, with a precision which is acceptable according to the modeling-estimated intracerebral currents. Altogether, we provide here a procedure to simplify and make easily applicable RePE-tDCS, which enables efficacious personalized treatments.

## Introduction

Advances in analyzing structural and functional brain features using powerful non-invasive neuroimaging methods are nowadays yielding intriguing insights into brain circuits, their variability across individuals, and their relationship to behavior (Van Essen, 2013; Kopell et al., 2014). If properly exploited, this knowledge can enhance the neuromodulation efficacy of transcranial Electrical Stimulation (tES), and transcranial Direct Current Stimulation (tDCS) in particular, by tuning the dimension, position and shape of the electrodes to the brain networks of interest (Murphy et al., 2009; Tecchio et al., 2014; Galletta et al., 2015; Cancelli et al., 2016, 2017; Grimaldi et al., 2016).

Following the need to concentrate the stimulation on the entire bilateral primary somatosensory representation of the whole body (S1), we recently introduced a new MRI-based neuronavigated procedure to customize brain stimulation through the use of the Regional Personalized Electrode (RePE) (Tecchio et al., 2013). The higher efficacy of personalized vs. non-personalized electrode in targeting the whole region was demonstrated experimentally (Cancelli et al., 2015a), and further confirmed by a recent modeling study (Parazzini et al., 2017). RePE targeting S1 (RePE-S1) was employed in two randomized clinical trials (RCT) showing an efficacy of this personalized 5-day tDCS against fatigue in multiple sclerosis (MS), thus we called the treatment Fatigue Relief in Multiple Sclerosis, FaReMuS (Tecchio et al., 2014; Cancelli et al., 2017). In the 2014 group, the mean fatigue reduction was 28% of the baseline after Real stimulation and 8% after Sham, *p* = 0.016. In the 2017 group, the fatigue symptoms reduction were 42% after Real and 20% after Sham, *p* = 0.012. We observed 7 responders, defined as the patients who changed the fatigue level more or equal to 20% of her/his baseline, in the 2014 group, and 9 in the 2017 one. The relevance of the target selection to effectively relieve fatigue can be perceived from the fact that other two tDCS trials, in which every parameter but the electrode shape and position were identical to those of the efficacious FaReMuS neuromodulation (Tecchio et al., 2014; Cancelli et al., 2017), did not produce the desired effects (Ferrucci et al., 2014; Saiote et al., 2014).

RePE-S1 electrode is shaped according to the projection onto the scalp of the individual cortical folding of the Rolandic sulcus. The projection was obtained by a neuronavigated procedure as the minimal distance from the scalp of the central sulcus reconstructed from the individual brain MRI (Tecchio et al., 2013). We conceived that the treatment should target the entire body somatosensory representation, as both limbs are impaired in people with MS and typically both sides. For this reason we decided to target the entire Rolandic sulcus post-central wall (Fischl et al., 2004; Destrieux et al., 2010). For simplicity, we selected from the left to the right Sylvian sulci, including the secondary somatosensory representation. In fact, as discussed above our main aim was to avoid frontal motor areas, while a wider parietal involvement was acceptable consistently with the literature (Pellicano et al., 2010; Engström et al., 2013; Vecchio et al., 2017).

Despite the promising results in relieving fatigue in people with multiple sclerosis (Tecchio et al., 2014; Cancelli et al., 2017), the application of RePE is still difficult because of the burdensome and complicated set up, that requires an experienced operator for the manually neuronavigated shaping of RePE with the patient affected by multiple sclerosis present along the entire shaping procedure, and an accurate neuronavigated positioning at the beginning of every stimulation session.

The tDCS treatments typically require multiple sessions. In fact, the duration of tES treatments, with one stimulation per day, ranges from 2 sessions (against alcohol or smoke craving, Boggio et al., 2008; Fregni et al., 2008) to 30 sessions (to support motor function in stroke patients, Hesse et al., 2011). In clinical contexts, the 5 consecutive day application is common in several diseases, including depression (Fregni et al., 2006b; Nitsche et al., 2009), pain (Zhu et al., 2017), and stroke (Boggio et al., 2007). The possibility of a simple home treatment set-up, easily manageable by the patient without special assistance, would therefore greatly increase the impact of RePE.

By theoretical computational modeling analysis, we tested here the relevance of RePE shape and position in terms of the tDCS differential efficacy on S1 with respect to primary motor cortex (M1) using a personalized vs. a non-personalized electrode. Computational modeling exploits high resolution MRI and accurate brain models based on Finite Element Method (FEM) to predict the electric field induced in each voxel of the brain during tDCS, thus allowing for an optimization of the tDCS montage, i.e., the maximization of the current flowing in the target region with respect to the other brain areas; in other words the maximization of the target region dosage (Antal et al., 2017).

We first aimed here to present and test a new MRI-based procedure to shape a RePE without the need for the neuronavigation technique, therefore bypassing the simultaneous presence of the patient and the experienced operator. Furthermore, we present a positioning technique that takes advantage of the scalp-space distances calculated on the 3D MRI reconstruction of the subject's head.

In the perspective of applying FaReMus at people home, we had here the second aim of introducing a procedure for easy and accurate repositioning of the electrode in a domiciliary environment (Pérez-Borrego et al., 2014; Charvet et al., 2015). We developed an adjustable helmet frame (AHF) to maintain the position of RePE during stimulation, and to allow an easy repositioning of RePE in multisession tDCS treatments even at the patient's home.

## Methods

The whole procedure was tested on 14 healthy volunteers (10 females, 4 males; age range 25–56 years, mean age 33.9 ± 11.0 years), eligible to the study in absence of clinical evidence or history of neurological or vascular diseases and with no pharmacological treatments. All participants provided written informed consent for participation in the study, as approved by the “Lazio 1-San Camillo Forlanini” Ethics Committee.

Each subject underwent a structural brain MRI exam with a 1.5 T scanner (Achieva, Philips Medical Systems, Best, The Netherlands). The acquisition protocol consisted of one 3D high resolution anatomical sequence empirically optimized to increase gray/white matter image contrast (T1-weighted Turbo Field Echo TR/TE/FA = 9.5 ms/4 ms/8°; 2,562 matrix resulting in an in-plane resolution 0.98 × 0.98 mm, slice thickness 1.2 mm, 160 coronal contiguous slice).

### Computerized RePE-S1 shape

As a preliminary step, we manually traced the central sulcus on the standard MNI152_T1_1 mm_brain MR template, thus avoiding the use of neuronavigation in shaping the RePE-S1. A total of 123 points were selected in the middle of the pre- and post-central sulcus (CS) walls, one for each of the 123 sagittal slices passing across the template central sulcus. These points span from the Sylvian sulcus of the left hemisphere to the same sulcus of the right hemisphere (MNI_CS).

**Figure 1:**
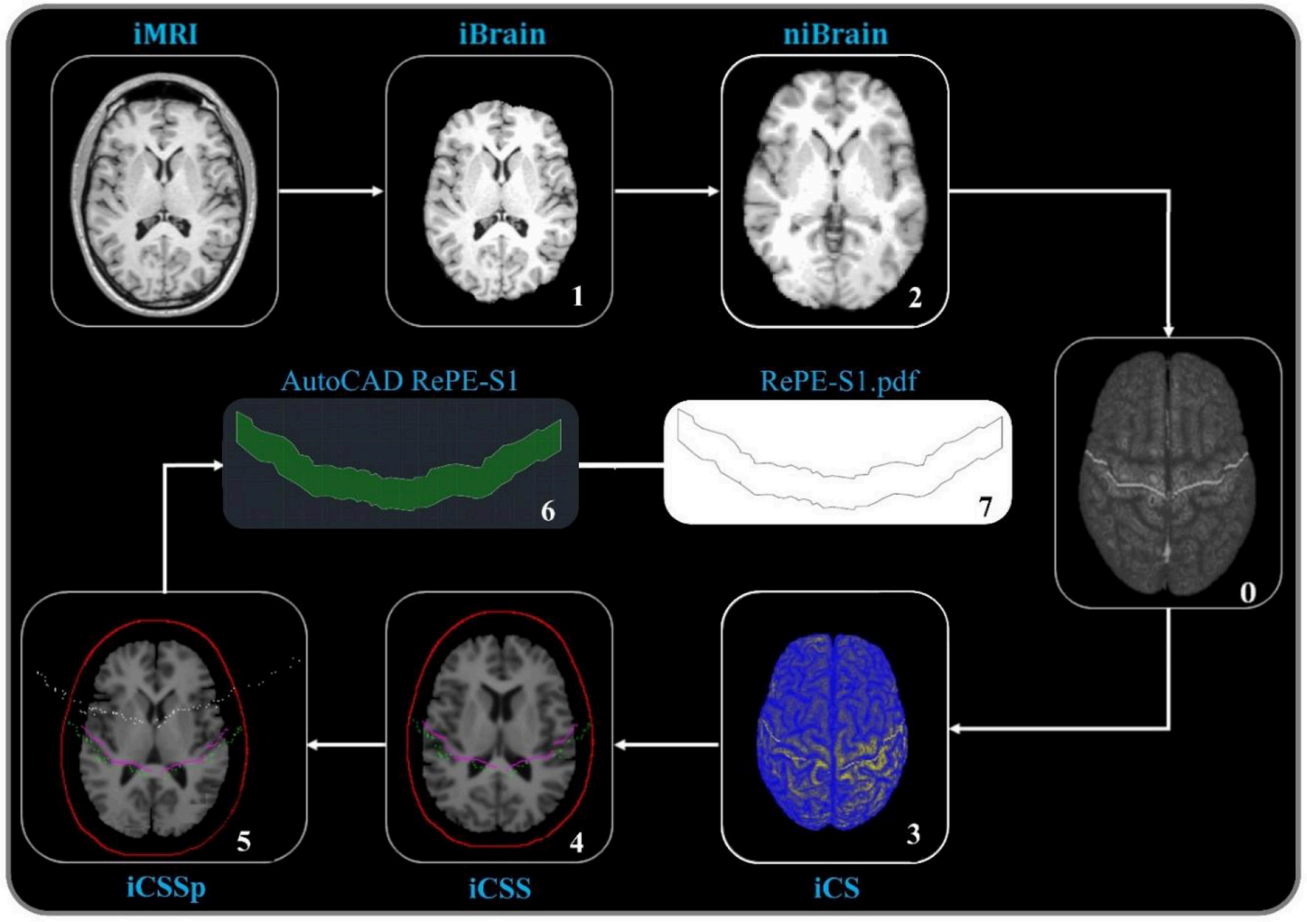
Computerized procedure to shape RePE. As detailed in the Methods, the steps from the individual brain MRI (iMRI), with segmentation of the brain (iBrain, 1), transformed into MNI standard model (niBrain, 2), where the central sulcus identified points are stored (once for every person, thus no number in the process applied in individual subject, 0) and retro projected obtaining the individual central sulcus (iCS, 3), projected on the scalp (iCSS, 4), and further projected on a planar surface (iCSSp, 5) so that by AutoCAD the RePE area is obtained (6) and printed (7).

The computerized process for shaping the individual RePE-S1 electrode (Figure 1) from her/his brain MRI consisted of the following steps:

1. Scalp and brain tissues were segmented from individual original MRIs (iMRI) using the Brain Extraction Tool (BET) of FSL (iBrain and iScalp);
2. iBrain images were spatially normalized with respect to the standard MNI152_T1_1 mm_brain MR template by means of the FMRIB's Linear Image Registration Tool (FLIRT) and the related transformation was saved as a matrix (TM);
3. The 123 MNI_CS points were automatically mapped onto the iMRI of each subject using the inverted spatial normalization matrix [TM^−1^, convert xfm & img2img] to obtain the 123 points on iMRI (iCS);
4. The iCS was projected onto the external surface of the 3D iScalp using an in-house software (iCSS), applying the minimal distance brain-scalp tissues for each point.
5. The iCSS were then projected to a plan, performing the iCSS on a plan (iCSSp).
6. RePE-S1 was designed using AutoCAD software (Autodesk AutoCAD 2017). The iCCSp coordinates were imported into the software in the native space and replicated at a distance of 2 cm from the original trace. Then, the two 2D iCCS were connected laterally to form a polygon.
7. The shape of RePE was exported in the PDF format and printed to be cut into sponge or conductive silicon electrodes.

### RePE positioning based on the 3D MRI head model

**Figure 2:**
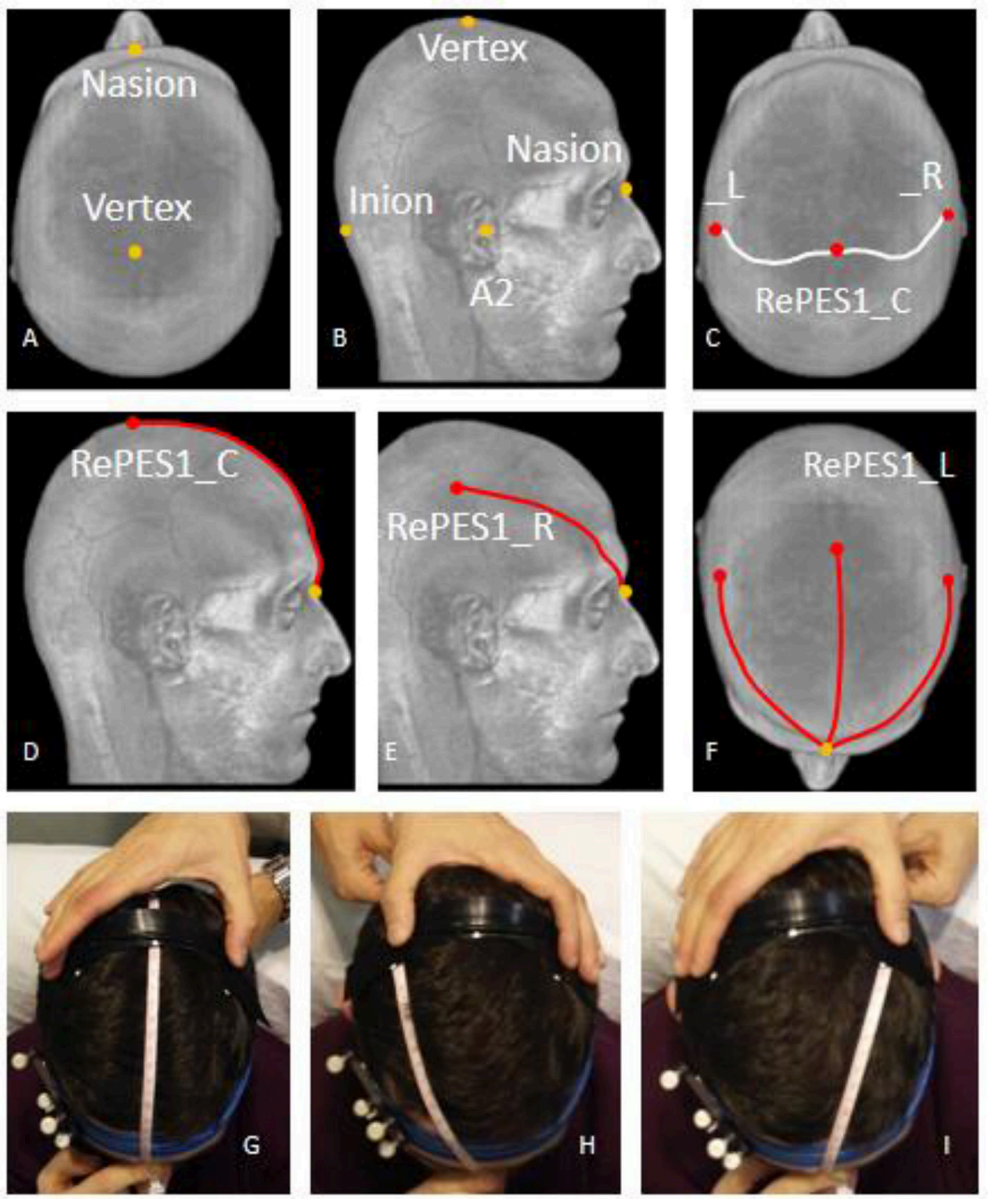
RePE positioning with 3D MRI-derived head space distances. As detailed in the Method section, representation of the curves on the scalp **(A–F)**, whose lengths derived from the individual 3D head model allow positioning RePE **(G–I)**.

To position RePE-S1 over the scalp of each subject in a way that the electrode would overlap her/his iCCS coordinates (minimal distance to the target region), we developed an MRI-based procedure to place each RePE-S1 in relation to the selected external landmarks on the subject's scalp. In particular, we used the following individual head points: Nasion (N, Figure 2A); Inion (I, Figure 2B); the central and lateral points of RePE-S1 located on the iCSS (RePE-S1_C, RePE-S1_L, RePE-S1_R, Figure 2C) with RePE-S1_C positioned on the line connecting Nasion and Inion. Then, over the 3D-rendered head, we measured the distances between N and the above defined three points: N-RePE-S1_C, N-RePE-S1_L, N-RePE-S1_R (Figures 2D–F). Given these three values, an operator can position RePE-S1 using the following procedure. She/he should place the zero (0) of a flexible meter on N and extend the meter to I (Figure 2G). Then he/she can center RePE-S1_C at distance N-RePE-S1_C from N, along the N-I line. Then he/she can adjust the RePE orientation through the N-RePE-S1_L and RePE-S1_R distances (Figures 2H,I).

### An adaptable helmet frame [AHF] for the electrode repositioning in multisession tDCS treatment

**Figure 3:**
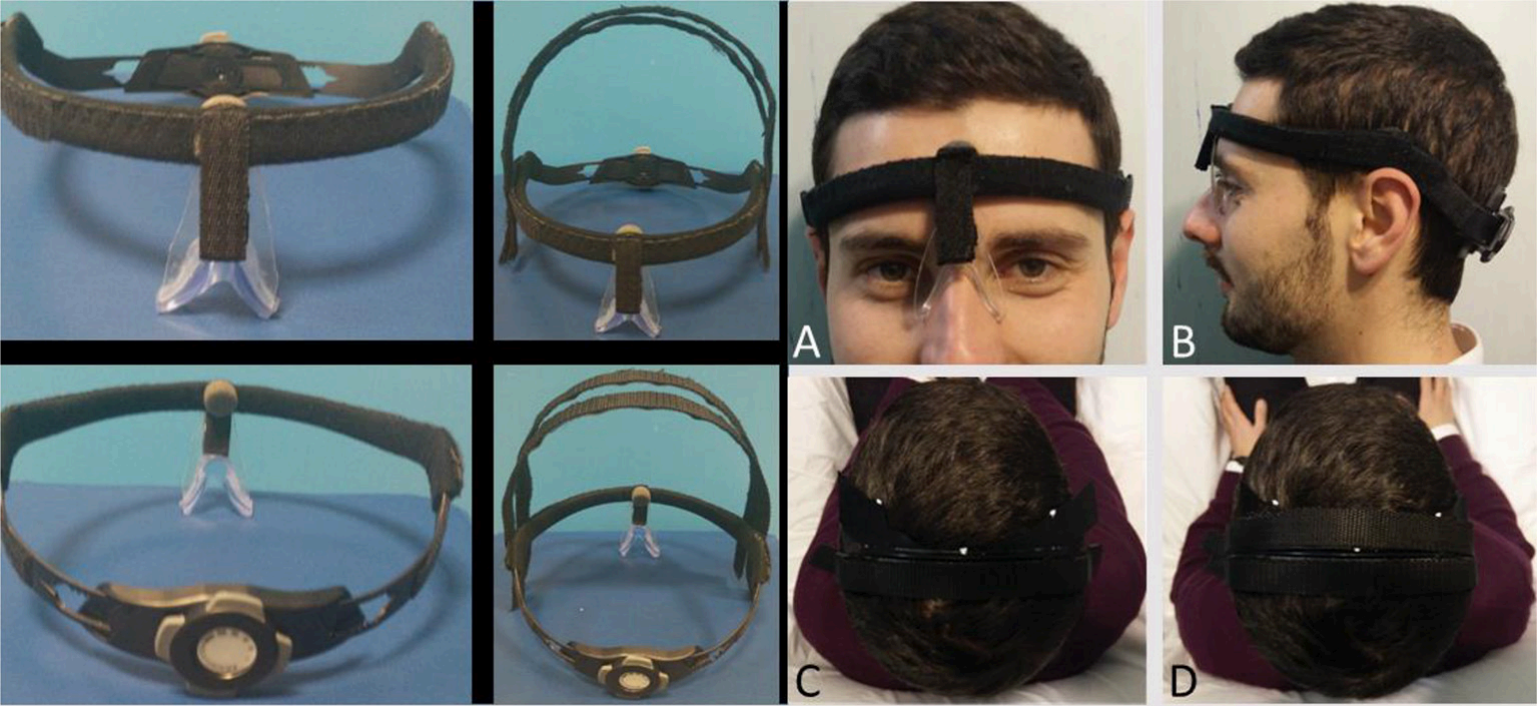
AHF for multisession RePE repositioning. Left: Adaptable Helmet Frame (AHF) without (left) and with (right) the Velcro strips to fix RePE. The occipital cathode electrode is fixed under the adjustable mechanism. Right: As detailed in the Methods, steps **(A,B)** allow positioning and re-positioning the AHF in same position. **(C,D)** fix the RePE position with respect to the AHF. Informed consent was obtained from the participant wearing the AHF to publish the image.

We developed an Adaptable Helmet Frame (AHF) using a plastic cyclist helmet (Figure 3). The helmet frame was equipped with an adjustable mechanism to comfortably fit the individual head of the subject, and it was integrated with a nasal support and Velcro bands to firmly fix the electrodes in the desired position.

Once RePE has been properly positioned, the operator can fix the AHF taking care not to move RePE electrode. In detail, (1) she/he positions the AHF so that the nasal support rests well on the nose (Figure 3A) and the inferior border lays over the top of the ears (Figure 3B). In this way, the AHF is always set in the same position. Thereafter, she/he (2) adapts the circumference to the individual head using the adjustment mechanism, and (3) fixes the electrode to the AHF stably with the two coronal Velcro strips (Figures 3C,D).

### Neuronavigated procedure to evaluate the RePE positioning and re-positioning

**Figure 4:**
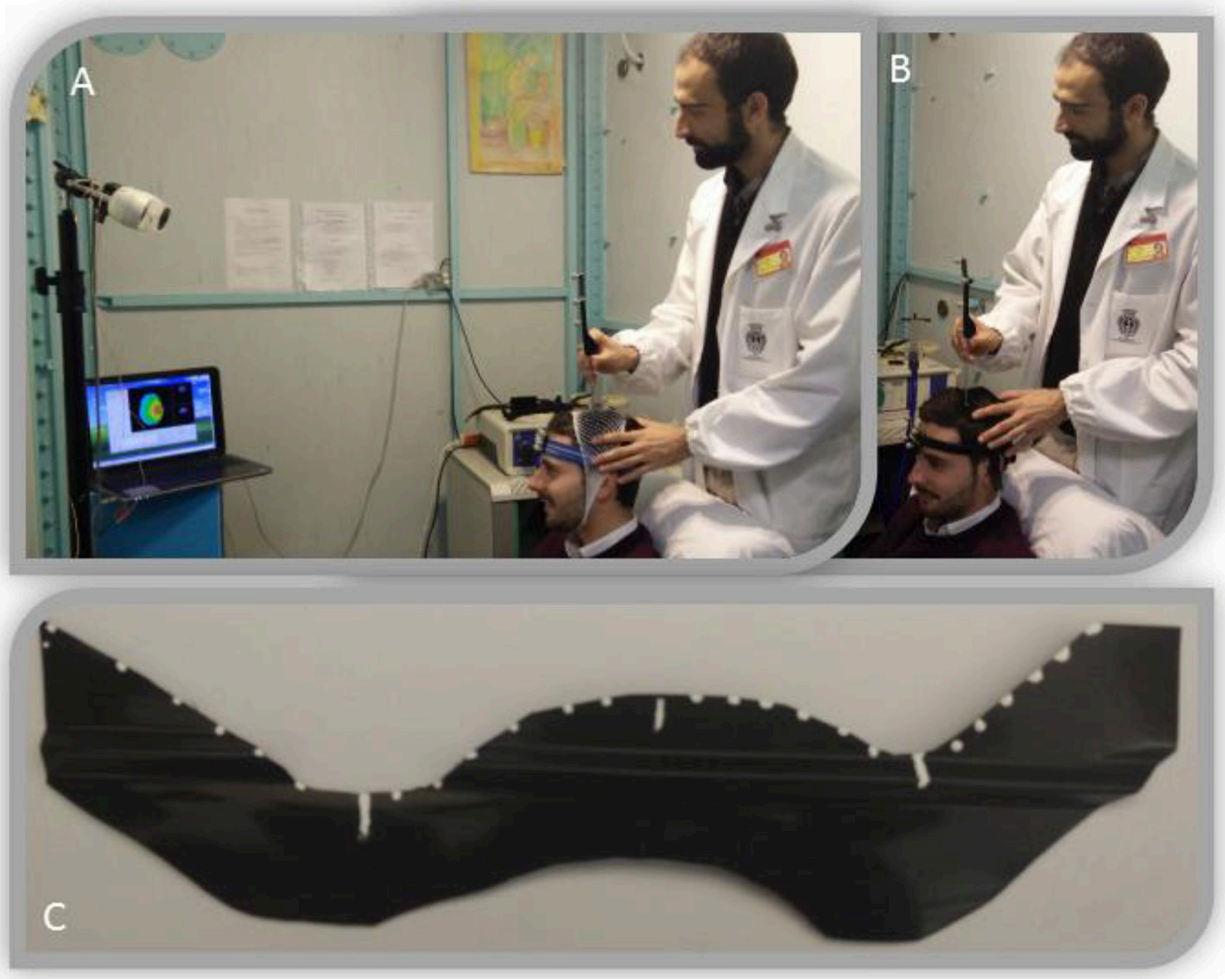
Neuronavigation setup used to estimate RePE positioning systems. Top: As detailed in the Methods, experimental neuronavigated procedure to test the RePE positioning via the MRI-derived 3D-head model measures **(A)** and via the AHF **(B)**. Informed consent was obtained from the participants executing the procedure and wearing RePE to publish the image. **(C)** RePE with the testing point used for the neuronavigated procedure.

We used neuronavigation (SofTaxic Neuronavigation System ver. 2.0, www.softaxic.com, E.M.S., Bologna, Italy) to test the repeatability of the RePE positioning based on the 3D MRI head model (Figure 4A), and to test the stability of the RePE AHF repositioning (Figure 4B).

We marked 27 points along the entire length of the electrode: the central point, 13 points on the left and 13 on the right of the central point (0.5 cm between points, RePEpoints, Figure 4C).

We collected the coordinates of RePEpoints in the individual MRI space, after positioning the electrode along the central sulcus projected on the scalp using the 3D-head space anatomical landmarks system, and repeated the procedure five times. For comparison, we also collected the RePEpoints positions when positioning RePE using the neuronavigated procedure. Finally, we calculated the distances between the corresponding RePEpoints and the points of the central sulcus projected onto the scalp (Figure 1).

To test the repeatability of the repositioning tool, we properly fixed the electrode into the AHF, we digitized the RePEpoints coordinates, and then we removed the AHF. We repeated the digitization of the RePE position after wearing AHF over 5 consecutive days.

### Statistical analysis

#### Evaluation of the identification of the computerized individual central sulcus

To evaluate the ability of the computerized procedure to locate the individual central sulcus, we compared the 123 iCSS points with those obtained by manually tracing the central sulcus on individual MRIs projected on the scalp (Manual iCSS).

Two independent researchers (a neuroradiologist—DL and a biomedical engineer with specific training in MRI data analysis—AC) selected every sagittal image where the central sulcus appeared on the individual segmented brain and recorded one point (X, Y, Z) for each slice. Accordingly, the number of points of Manual iCSS varied across subjects between a minimum of 102 and a maximum of 117. We projected the Manual iCS onto the external surface of the 3D iScalp using the same software that was used for the Computerized procedure (Manual iCSS).

We quantified the accuracy of the Computerized iCSS identification as the point-to-point Euclidean distance to the Manual iCSS set (nearest points) and the Intra-Class Correlation (ICC) between Computerized iCSS and Manual iCSS.

#### RePE position accuracy

We evaluated the repeatability of RePE position using the Euclidean distances of the RePEpoints from the corresponding projections on the scalp of the central sulcus points. In order to detect possible systematic positioning errors, we submitted the differences of the 3D coordinates (x, y, z) for the 27 points in successive positions to an analysis of variance (ANOVA) with the Positioning Error (Rep2-1, Rep3-1, Rep4-1, Rep5-1) and Positioning Method (Neuronavigation, 3D MRI-head distances) as within-subject factors. Any systematic displacement will correspond with significant Positioning Error effect. We performed all statistical analysis using SPSS (IBM).

### Intracerebral current induced by RePE: MRI-derived finite element method

The electric field (EF) distributions induced into the brain by RePE-S1 and non-RePE were computed using a FEM forward model. Several configurations were analyzed: different positions of RePE-S1 and different return electrode locations. One MRI scan of a 39 year old adult male head was segmented using an algorithm developed in-house for automated tissue compartment segmentation (Huang et al., 2013). Adaptive volumetric meshing was applied to the tissue segmentation in ScanIP (Simpleware Ltd, Exeter, UK) with a compound coarseness of −15 (maximum edge length 1.85 mm, target minimum 0.775 mm, target Jacobian minimum 0.1). The resulting meshes consisted of >10^7^ quadratic tetrahedral elements and >1.5·10^6^ degrees of freedom. Further refined meshes were found to have no noticeable effect on simulation results. The mesh was imported into COMSOL Multiphysics 4.3 (Burlington, MA) to simulate quasi-static volume conductor physics, since this approximation is acceptable for tES (Smith, 1992; Nathan et al., 1993; Eshel et al., 1995). The overall volume was divided in seven compartments with different electrical conductivity (Datta et al., 2011; Parazzini et al., 2011; Wagner et al., 2014; Galletta et al., 2015; Cancelli et al., 2016) representing: gray matter (σ = 0.276 S·m^−1^), white matter (σ = 0.126 S·m^−1^), cerebral spinal fluid (CSF, σ = 1.65 S·m^−1^), skull (σ = 10^−2^ S·m^−1^), air (σ = 10^−4^ S·m^−1^), fat σ = 0.025 S·m^−1^), and skin (σ = 0.465 S·m^−1^). Two additional masks including the Post and Anterior Central Gyruses were created and intersected with gray matter to segment the Primary Somatosensory and Motor Area, respectively, for a quantitative evaluation of the electric field generated at the target. The electrodes were imported into ScanCAD (Simpleware Ltd, Exeter, UK) for manual positioning over the scalp of the 3D model. During transcranial electrical stimulation (tES), a conductive gel (σ = 0.3 S·m^−1^) is interposed between the silicon electrode and the scalp. The adopted model was modified accordingly.

Within COMSOL, the Laplace equation ∇(σ∇V) = 0, with σ being the electric conductivity and V the electric potential, was solved considering the Neumann boundary conditions. COMSOL implemented a linear system solver of conjugate gradients with a relative tolerance of 10^−6^ mA of current stimulation which was applied for all the montages, so a boundary condition of orthogonal current density was implemented to the anode and the cathode. For each electrode, the current value was divided by the skin-electrode contact area and was applied as a current density boundary condition and assigned to the mesh nodes as current loads representing the right-hand-side of the linear system of equations.

#### RePE-S1 vs. Non-RePE-S1 bilateral stimulation

**Figure 5:**
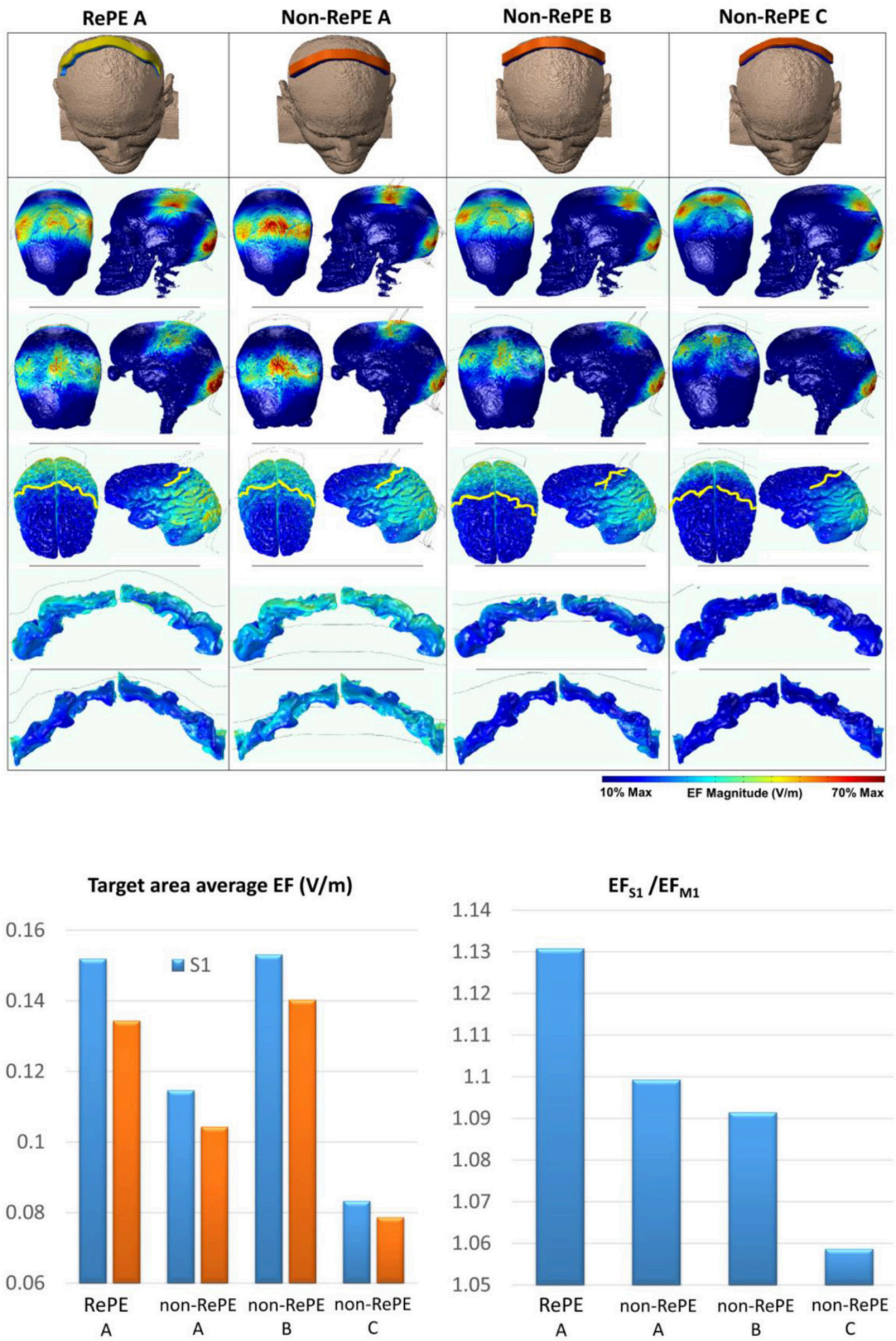
Modeling: RePE-S1 vs. non-RePE-S1 comparison. Top: First row: The four anodal electrodes tested against the same reference with twice the RePE's area positioned on Oz: personalized S1 electrode positioned 0.5 cm frontal to the scalp projection of the central sulcus (RePE A), the non-personalized strip shaped to overlay Cz and from C3 to C4 (Non-RePE) with a part on hand/mouth S1 (A), on Cz (B), or on CPz (C). From the second row: electric field amplitude (EF) on the scalp (second row), dura mater (third row), cortical surface (fourth row), and S1 post-central (fifth row) and M1 pre-central gyri (sixth row). Bottom: Average EF (V/m) in the whole body S1 (light blue) and M1 (orange) for the four montages (Left) and the ratio in S1 with respetct to M1 (Right).

Two different electrodes of 35 cm^2^, RePE over S1 and a non-personalized electrode (semi-curved pad 17.5 × 2 cm, Cancelli et al., 2015a; Parazzini et al., 2017) placed in three different locations were examined as anodes (Figure 5). A standard electrode with twice the area of the anodes [70 cm^2^, in order to decrease the current density under the return electrode (Tecchio et al., 2014, 2015; Cancelli et al., 2015a,b, 2017)] was used as a cathode centered over Oz.

We examined four montages:

- Montage RePE A: RePE-S1 designed and positioned over S1 as detailed in sections Computerized RePE-S1 shape and RePE Positioning Based on the 3D MRI Head Model;
- Montage Non-RePE A: non-RePE over hand/mouth S1;
- Montage Non-RePE B: non-RePE centered on Cz of the 10–10 International System (IS);
- Montage Non-RePE C: non-RePE centered on CPz of the IS.

#### Reference electrode evaluation

**Figure 6:**
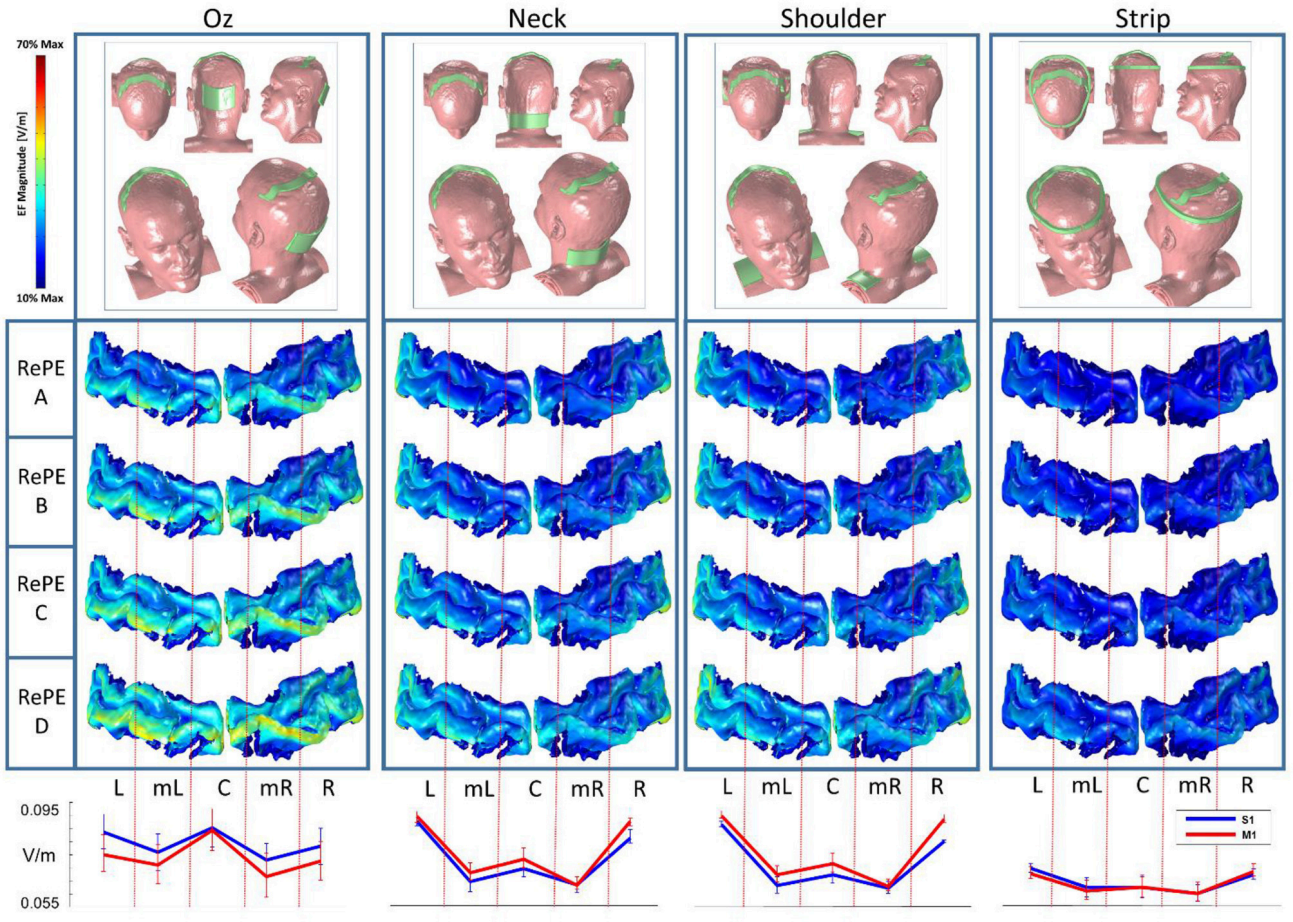
Modeling: Reference evaluation in RePE tDCS. **(Top)** For each of the reference electrode position (Oz, neck, shoulders, and a ring centered on RePE) the electric field amplitude (EF) on the cortical strip including the S1 and M1 cortical gyri for the four RePE positions: with the anterior border fitting the CS scalp projection (RePE A), 0.5 cm (RePE B), or 1 cm (RePE C) and 1.5 cm ahead (RePE D). **(Bottom)** Average EF (V/m) in the five sections (Left L, medio-Left mL, central C, medio-Right mR, and Right R) of S1 (blue) and M1 (red), averaged across the RePE positions, for the four reference electrodes.

Following the results of the montages described in section Reference Evaluation in RePE tDCS, no more computations involving either the non-RePE anode or positions behind the Central Sulcus of RePE-S1 were run. Sixteen montages with RePE-S1 were evaluated with the aim of optimizing the RePE stimulation: four different reference electrodes (pad over Oz, Neck, Shoulders and a strip around the head, Figure 6), each with four different positions of RePE-S1, with the anterior border of RePE-S1 overlapping the CS scalp projection (RePE A), 0.5 cm ahead of the CS scalp projection (RePE B), or 1 and 1.5 cm ahead (RePE C and RePE D).

## Results

### Computerized RePE design

#### Computerized individual central sulcus on the scalp

The mean average distance across points and subjects of iCSS and manual iCSS sets was 2.9 ± 3.2 mm, and the mean intra-class correlation was 0.999 ± 0.0013 (ICC).

### RePE positioning and re-positioning

#### RePE position based on head fiducials

The coordinates of the RePE position did not differ between neuronavigated positioning and new computerized positioning based on the distances derived from the 3D-head model, as indicated by the *Positioning Method* factor [*F*_(3, 3)_ = 0.263, *p* = 0.849]. The mean repetition error in terms of the Euclidean distance was 3.0 mm for neuronavigation and 3.5 mm for anatomical landmark-system across all repetitions (Table 1). When compared with paired-sample *t*-test, no differences were found [*t*_(9)_ = −1.110, *p* = 0.296]. No systematic displacement occurred in any direction, as indicated by the *Positioning Error* factor [*F*_(9, 45)_ = 0.768, *p* = 0.646].

**Table 1:** RePE positioning via 3D head MRI distances vs. Neuronavigation.

**Session**	**Neuronavigation (mm)**				**3D head model MRI distances (mm)**			
	**Distance**	***X***	***Y***	***Z***	**Distance**	***X***	***Y***	***Z***
1	3.1 ± 1.4	1.1 ± 2.9	0.5 ± 2.8	−0.8 ± 2.7	3.7 ± 0.6	1.9 ± 2.7	−3.1 ± 4.1	2.0 ± 3.9
2	2.6 ± 1.5	0.4 ± 3.0	−0.6 ± 3.1	−0.4 ± 2.2	2.5 ± 0.5	1.5 ± 2.7	−2.3 ± 4.3	2.8 ± 4.9
3	2.7 ± 1.3	0.9 ± 2.1	−1.9 ± 1.6	0.7 ± 2.3	3.7 ± 0.5	−2.0 ± 3.7	2.3 ± 4.4	0.9 ± 3.7
4	3.4 ± 1.1	1.4 ± 3.3	0.0 ± 2.2	0.5 ± 2.4	4.2 ± 0.7	2.0 ± 3.0	2.0 ± 2.7	−3.9 ± 2.6
5	3.0 ± 0.6	1.3 ± 2.7	−1.4 ± 4.2	1.2 ± 3.6	3.8 ± 1.2	0.8 ± 3.5	−0.2 ± 4.6	2.5 ± 5.0
Overall	3.0 ± 1.2	1.0 ± 2.8	−0.7 ± 2.8	0.2 ± 2.6	3.5 ± 0.8	0.8 ± 3.3	−2.8 ± 4.3	0.3 ± 2.1

*Mean displacements between each of the 27 RePEpoints in each electrode and the corresponding point of the scalp projection of the central sulcus, both as Euclidean distance (Distance) and the differences of each of the three Cartesian coordinates (x, y, z), when the personalized electrode was positioned either using the neuronavigated procedure or the distances derived from the 3D-head model from individual MRI*.

#### Adaptable helmet frame [AHF] for the electrode repositioning in multisession tDCS treatment

The mean repositioning error in terms of the Euclidean distance was 4.8 ± 1.1 mm across all repetitions and subjects. The ANOVA design analyzing the coordinate differences displayed that no systematic displacement occurred in any direction (Table 2). In fact, *Positioning Error* factor was [*F*_(3, 24)_ = 0.086, *p* = 0.967].

**Table 2:** RePE position by adaptable helmet frame [AHF].

**Day**	**Distance**	***X***	***Y***	***Z***
1	4.8 ± 1.4	−2.0 ± 1.9	0.9 ± 1.4	0.8 ± 1.2
2	4.9 ± 1.1	−0.7 ± 3.6	3.0 ± 3.1	−1.0 ± 2.4
3	4.7 ± 1.0	0.8 ± 1.2	2.8 ± 0.4	0.7 ± 2.3
4	4.9 ± 1.1	−2.1 ± 2.8	0.6 ± 3.8	1.1 ± 2.4
5	4.6 ± 0.7	−2.0 ± 3.9	0.8 ± 4.2	0.5 ± 2.8
Overall	4.8 ± 1.1	−0.7 ± 2.7	1.6 ± 2.6	0.4 ± 2.4

*Mean displacements between each of the 27 RePEpoints in each electrode positioning with the AHF and the corresponding point of the scalp projection of the central sulcus, both as Euclidean distance (Distance) and the differences of each of the three Cartesian coordinates (x, y, z)*.

### Electric field distribution analysis

#### RePE-S1 vs. Non-RePE-S1 bilateral stimulation

The different shape and position of the electrode used as anode and cathode (Figure 5) played a key role in determining the boundaries of the cortical area that was stimulated. Specifically, RePE A generated a cortical EF diffused uniformly from the central sulcus to the occipital area, position of the return electrode. Therefore, the stimulation affected the entire S1 with an average EF (EF_AVE_) of 0.152 V/m for the 1.5 mA/35 cm^2^ current density, while the EF was minimized in M1 (EF_AVE_ = 0.134 V/m). In the montage non-RePE A the non–personalized electrode was placed ahead of the Central Sulcus and the stimulation involved both the entire S1 (EF_AVE_ = 0.153 V/m) and M1 (EF_AVE_ = 0.140 V/m). The montage non-RePE B generated a similar EF magnitude to that of RePE A in the central section of S1, but decreasing toward the Sylvian Fissure (Figure 5). This is conceivably because the non-personalized electrode didn't follow the convolutions of the entire Post Central Gyrus. Thus, the resulting average EF in S1 was lower than RePE A, EF_AVE_ = 0.114 V/m. The montage non-RePE C was placed posteriorly to S1 so the stimulation barely affected both S1 (EF_AVE_ = 0.083 V/m) and M1 (EF_AVE_ = 0.078 V/m).

The maximum ratio between the EF induced in S1 and M1, was obtained by montage RePE A (EF_AVE_^S1^ > 13% EF_AVE_^M1^).

#### Reference evaluation in RePE tDCS

The computational analysis using various positions of the RePE anode with different cathodes revealed that the highest efficacy is obtained using a standard reference electrode on Oz, placed oppositely to the area in which the induced EF is required to be low (in this case, M1). The average EF obtained with all the RePE positions with Oz reference (Figure 6 bottom, Table 3) is the highest (0.91 V/m) in the Left, Central and Right part of S1, even if the effect in the central part is similar in M1. Indeed, the other reference choices cannot discriminate between the EF in M1 and S1 at all. In particular the Neck and Shoulder reference induced maximal EF only in the lateral parts (L and R, 0.09 V/m) of the target region. The strip reference causes a low EF from Left to Right both in S1 and M1 (0.58 V/m).

**Table 3:** Reference evaluation in RePE tDCS.

**Montage**	**Oz**					**Neck**					**Shoulders**					**Strip**				
	**L**	**mL**	**C**	**mR**	**R**	**L**	**mL**	**C**	**mR**	**R**	**L**	**mL**	**C**	**mR**	**R**	**L**	**mL**	**C**	**mR**	**R**
RePE A	1.135	1.090	1.029	1.144	1.101	0.993	0.945	0.960	0.991	0.967	0.974	0.942	0.946	0.992	0.923	1.071	1.038	1.008	1.003	1.006
RePE B	1.139	1.091	1.026	1.124	1.093	0.994	0.964	0.960	1.026	0.932	0.977	0.948	0.950	1.006	0.916	1.065	1.056	1.016	1.026	0.983
RePE C	1.126	1.076	1.022	1.103	1.094	0.991	0.967	0.961	1.024	0.929	0.974	0.952	0.952	1.008	0.912	1.057	1.056	1.011	1.022	0.980
RePE D	1.106	1.062	1.011	1.061	1.073	0.981	0.962	0.960	1.006	0.919	0.966	0.950	0.952	0.996	0.902	1.036	1.040	1.000	0.991	0.964

*Electric field in S1 and M1 ratio delivered by the 16 montages in the five different portions of the target regions*.

Comparing the positions of the RePE anode over the scalp, best efficacy is obtained by the RePE A position (EF_S1_/EF_M1_ = 1.13). RePE B, with a slip of 5 mm from Position A toward the projection of M1, generated similar EF at the target (EF_S1_/EF_M1_ = 1.12), while RePE C and RePE D (1 and 1.5 cm form Position A) significantly decreased performances.

## Discussion

The main achievement of this study is the double development of an easy and effective tool to shape and position a tES electrode personalized for stimulating a specific cortical district, and for repositioning it for a multi-sessions protocol, specifically designed to be used by the patient at home.

When the stimulation target is a limited cortical area, modeling suggests that a ring configuration of HD electrodes is the most appropriate montage for focusing current into such area (Datta et al., 2009; Cancelli et al., 2016). On the contrary, the idea of the RePE-tDCS came out from the necessity to uniformly stimulate an extended cortical region (S1) and to minimize the degree to which EF was induced in the adjacent region (M1). The present procedure makes this personalized stimulation more easy and affordable.

The results obtained through computational modeling support the idea that RePE-tDCS is a valid montage for differentiating the tDCS dose between two contiguous areas. A non-RePE electrode cannot reach the same efficiency in terms of local specificity of the induced EF, regardless of its position on the scalp. On the other hand, it is clear that in any standard tDCS montage, i.e., with 2 electrodes, the position of the second electrode is crucial in managing the direction of the current flow. Because of the high conductivity of the CSF, the EF generated by tDCS is diffused between the two electrodes. Therefore, the electrode shape is not the unique parameter which should be considered in a RePE-montage optimization. To realize a correct RePE-tDCS, the shape and position of the reference electrode are essential (Figure 6). In both extra-cephalic standard electrode references—neck and shoulders—and a strip around the head, the current flow did not affect the cortex as much as utilizing an occipital electrode. The four positions used to test RePE suggest that the tDCS dose is more differentiated between the two areas when the edge of the electrode, opposite to the reference, is close to the boundary delimiting the two areas (RePE A). On the other hand, a similar EF magnitude is obtained using a position of the anode that is 0.5 cm away (RePE B). This suggests that a position displacement of < 5 mm does not affect the stimulation.

The computerized procedure we developed to design RePE identifies the edge of the target area on the MNI model and retro-transforms it onto the individual MRI coordinates. We evaluated the accuracy of this step through a control test, and we observed errors to the order of 2–3 mm with respect to the visual inspection of manual tracing, as tested by two independent MRI analysis experts.

The computerized procedure, processing the individual MRI using standard well-tested software, offers multiple advantages relative to manual protocols including:

(1) a significant time reduction in the electrode preparation; (2) no need to have the patient in the laboratory for the RePE preparation; (3) no need for a neuronavigation system; (4) the reduction of the variability in the RePE shape, depending on the experience of the researcher or technician who prepares the electrode.

In the present investigation, we used a 3D T1 sequence (MPRAGE) on a 1.5 T MRI scanner to obtain anatomical scans. In some experiments we tested lower resolution images in order to consider the minimal requirement of the quality of scans necessary to apply the proposed computerized procedure. The scans do not require high-resolution 3D sequences, but the sulcus can be well identified using almost any acquisition sequence (i.e., T1, T2, Flair) in any direction axial, sagittal and coronal, with a voxel resolution around 1 mm^2^ and maximum 3 mm of slice thickness.

The present paper provides a simple and low-cost procedure for the tES electrode positioning and re-positioning (AHF), using easily recognizable anatomical scalp landmarks in any tES multisession treatments. Being a non-invasive brain stimulation, tDCS painlessly delivers electrical current of low intensity through the skull to selected areas of the brain (Nitsche et al., 2000; Antal et al., 2017), and offers a very high safety profile. Several double-blind studies proved multisession tDCS treatments to have negligible side effects offering an analgesic effect against chronic pain (Soler et al., 2010; Brietzke et al., 2016), to relieve symptoms in attention deficits and depression (Gögler et al., 2016; Cachoeira et al., 2017), in addition to relieving fatigue in multiple sclerosis. The suitability and repeatability consistent with those obtained via neuronavigation further simplify tES procedures, making them largely suitable for multisession home treatments and clinical protocols (Pérez-Borrego et al., 2014; Amatya et al., 2015; Charvet et al., 2015).

The test of the whole procedure is performed only for the single post-central gyrus RePE shape and position. This is a limitation of the present study. However, it is conceivable that the presented procedure can be easily exported to other extended cortical targets, like (almost) all bilateral associative cortices. While in the case of the central sulcus we proved that our procedure was appropriate, for different sulci that show greater variability between individuals other brain normalization procedures that take into account all cortical folding (Destrieux et al., 2010) might be evaluated. In the present work, we developed an automatized procedure to shape and position an electrode to focus the neuromodulation effect on a cortical area, limiting as much as possible the direct effects on contiguous cortical areas. We developed this procedure, based on individual brain MRI, since we documented that the personalization of the electrode is necessary to modify the excitability of the entire area of the postcentral gyrus from the left to the right Silvian sulcus (Cancelli et al., 2015a).

Future perspectives in the field of transportation research will consider to build and strengthen easy to use methods for other personalized tES. The relevance of the homologous areas balance in different neurological and psychiatric disorders (Fregni et al., 2006a; Ferrucci et al., 2009; Nitsche et al., 2009), and the indication that equal bilateral stimulation impinge positively counteracting pathological imbalances (Pahor and Jaušovec, 2018; Tseng et al., 2018), suggests that developing electrodes to target such bilateral representations will be more frequent in future. Conceivably, to build electrodes that take into account the specific individual cortical folding is an advantage in focusing the stimulation of high-definition tES (Edwards et al., 2013; Moreno-Duarte et al., 2014; Malavera et al., 2015; De Ridder et al., 2017). In fact, we devoted this work to develop an easy applicable tool to stimulate a specific cortical region in individual patients, without the need of neuronavigation, and we underlined that other RePE to target other cortical folding can be shaped and positioned via the procedure offered in the present work. Nevertheless, specific cases require proper modeling analyses in selecting the proper montage, specifically defining the reference in use.

## Conclusions

We provide a model-based investigation of the RePE tDCS accompanied by a simple, low-cost procedure to easily apply it. We tested the technique for the stimulation of the primary somatosensory area, but the procedure, if supported by a correct application of the return electrode, can potentially be applied to any cortical region to differentiate the EF induced at the target region. Furthermore, as a complete and computerized design, positioning and re-positioning of the electrodes establishes a relevant advancement for any home tES treatment.

## Author contributions

FT and AC: contributed to conception and design of the study; AC, GA, CC, AG, and DL: acquired and analyzed the data; AC, FT, and VP: drafted and finally revised the manuscript and the figures.

### Conflict of interest statement

The authors declare that the research was conducted in the absence of any commercial or financial relationships that could be construed as a potential conflict of interest.
